# PMSA as a potential modulator of calcineurin phosphatase activity

**DOI:** 10.1038/s41598-026-48882-9

**Published:** 2026-04-22

**Authors:** Eunjin Cho, Seongmin Cheon, Dong Kyu Choi, Hee-Young Yang, Chungoo Park, Tae-Hoon Lee

**Affiliations:** 1 New Drug Development Center, Daegu-Gyeongbuk Medical Innovation Foundation (K-MEDI Hub), Daegu, 41061 Republic of Korea; 2 School of Biological Sciences and Technology, Chonnam National University, Gwangju, 61186 Republic of Korea; 3 School of Life Sciences, BK21 FOUR KNU Creative Bio Research Group, KNU G-LAMP Project Group, KNU Institute of Basic Sciences, College of Natural Sciences, Kyungpook National University, Daegu, 41566 Republic of Korea; 4 Preclinical Research Center, Daegu-Gyeongbuk Medical Innovation Foundation (K-MEDI Hub), Daegu, 41061 Republic of Korea; 5 Institute of Systems Biology & Life Science Informatics, Chonnam National University, Gwangju, 61186 Republic of Korea; 6 Department of Oral Biochemistry, Dental Science Research Institute, School of Dentistry, Chonnam National University, Gwangju, 61186 Republic of Korea; 7 Proteomics Core Facility, BioMedical Research Institute, Kyungpook National University Hospital, Daegu, 41940 Republic of Korea

**Keywords:** Biochemistry, Cell biology, Diseases, Drug discovery

## Abstract

Osteoporosis is a major skeletal disorder characterized by reduced bone strength and increased risk of fractures. Excessive osteoclast-mediated bone resorption is a primary cause of this condition, underscoring the need for effective anti-resorptive therapies. *N*-phenyl-methylsulfonamido-acetamide (PMSA) compounds have been previously identified as potential anti-resorptive agents that inhibit osteoclastogenesis. In this study, ribonucleic acid (RNA)-sequencing and proteomic analyses identified calcineurin (CaN) as a potential target of PMSA implicated in osteoclast differentiation. PMSA bound to CaN and suppressed its phosphatase activity, which is essential for the activation and translocation of nuclear factor of activated T cells, cytoplasmic 1 (NFATc1), a key regulator of osteoclastogenesis. PMSA treatment resulted in altered NFATc1-related signaling and increased phospho-NFATc1 levels in osteoclasts. Overall, these findings suggest that PMSA may inhibit CaN activity during osteoclast differentiation, positioning it as a promising therapeutic candidate for osteoporosis.

**Supplementary Information:**

The online version contains supplementary material available at 10.1038/s41598-026-48882-9.

## Introduction

Osteoporosis is a prevalent metabolic bone disorder characterized by progressive bone loss, leading to increased skeletal fragility and an elevated fracture risk^1^. With an aging global population, osteoporosis has become a significant public health concern. Although several antiresorptive medications have been developed to suppress osteoclast (OC) activity in patients with osteoporosis^2^, these drugs are associated with adverse effects and are unsuitable for prolonged use^3,4^.

Calcineurin (CaN) is a serine/threonine phosphatase that dephosphorylates its protein substrates^5^. It functions as a heterodimer comprising a catalytic A chain and a calcium-binding regulatory B chain^6^. CaN participates in various physiological processes, including immune responses, learning and memory, and cardiac hypertrophy, through interactions with multiple substrates^7–10^. Among its most studied targets are the nuclear factor of activated T cell (NFAT) proteins, which remain in a hyperphosphorylated, inactive state in the cytosol^11^. CaN dephosphorylates NFATs, enabling their nuclear translocation and activation of various transcription programs. During osteoclastogenesis, CaN is activated by increased calcium concentrations following receptor activator of nuclear factor kappa B ligand (RANKL) stimulation^12^. The calcium-sensing protein calmodulin (CaM) and the CaN B subunit form the CaM–CaN complex, which releases the autoinhibitory domain from the catalytic site of the CaN A subunit^11^. Activated CaN then dephosphorylates NFAT, cytoplasmic 1 (NFATc1), the master transcription factor driving OC differentiation, thereby promoting its nuclear localization^13,14^.

CaN inhibitors such as cyclosporin A (CsA) and tacrolimus (FK506) are clinically used immunosuppressants that prevent transplant rejection^15^. FK506 and CsA suppress CaN phosphatase activity by binding to their respective immunophilins, FKBP12 and cyclophilin. These immunosuppressants diminish immune responses by inhibiting pathways required for T cell activation. For example, CsA inhibits the synthesis of interleukins necessary for T lymphocyte differentiation^16,17^. Both CsA and FK506 inhibit OC differentiation in bone marrow-derived cells^18^. However, CsA has adverse effects, including gingival hypertrophy, tremors, hypertension, and hepato- or nephrotoxicity^19^.

It has been reported that *N*-phenyl-methylsulfonamido-acetamide (PMSA) compounds inhibit NFATc1 nuclear translocation during OC differentiation^20^. PMSA suppresses OC differentiation in bone marrow-derived macrophages (BMMs), reduces bone-resorptive activity, and attenuates bone loss in a mouse model of estrogen-mediated osteoporosis. In this study, the mechanism by which PMSA regulates NFATc1-mediated OC differentiation through integrated transcriptional and protein assays was further investigated.

## Results

### PMSA preferentially affects RANKL-mediated pathways over M-CSF-mediated processes

**Fig. 1:**
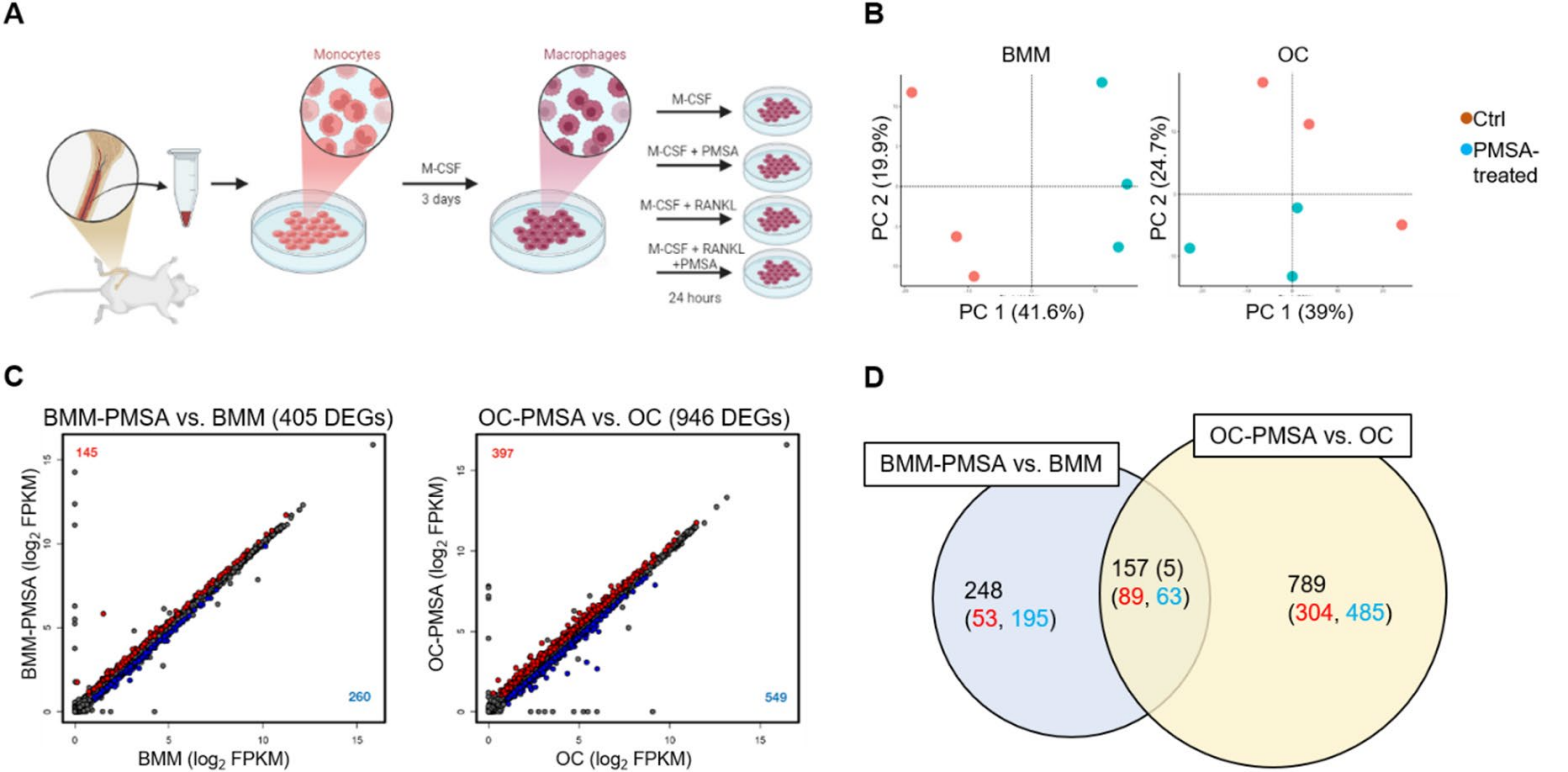
PMSA preferentially affects RANKL-mediated processes over M-CSF-mediated pathways. (**A**) Schematic representation of the four experimental groups analyzed by RNA-sequencing: BMM (M-CSF), BMM-PMSA (M-CSF + PMSA), OC (M-CSF + RANKL), and OC-PMSA (M-CSF + RANKL + PMSA). (**B**) Principal component analysis comparing PMSA-treated and untreated (Ctrl) samples in BMMs and OCs. All samples were analyzed in triplicate. (**C**) Scatter plots of DEGs. Significantly upregulated and downregulated genes are shown in blue and red, respectively; gray dots represent non-DEGs. (FPKM > 1, q-value < 0.05). (**D**) Venn diagram illustrating DEGs in BMMs and OCs. Five shared DEGs exhibit opposite expression patterns between the two comparisons.

Our previous study demonstrated that PMSA inhibits OC differentiation by blocking NFATc1 dephosphorylation^20^. To identify the direct targets of PMSA, ribonucleic acid (RNA)-sequencing was performed. Macrophage colony-stimulating factor (M-CSF) is required for macrophage differentiation and maintenance^21^. To evaluate the effect of PMSA during OC differentiation, four groups were analyzed: BMM (M-CSF), BMM-PMSA (M-CSF + PMSA), OC (M-CSF + RANKL), and OC-PMSA (M-CSF + RANKL + PMSA). Cells were treated with PMSA for 24 h before RNA extraction (Fig. 1A). Principal component analysis revealed distinct clustering following PMSA treatment, accounting for 61.5% of the variance in BMMs and 63.7% in OCs (Fig. 1B), indicating that PMSA alters gene expression in both cell types.

Differentially expressed genes (DEGs) were analyzed by comparing expression levels between PMSA-treated and untreated groups. The analysis revealed that PMSA upregulated 145 genes and downregulated 260 genes in BMMs (Fig. 1C). In OCs, the effect was more pronounced, with 397 genes upregulated and 549 downregulated—more than twice the number observed in BMMs—indicating preferential action on RANKL-dependent pathways.

Comparison of DEGs between BMMs and OCs revealed 405 DEGs in the M-CSF group and 946 DEGs in the M-CSF and RANKL-treated group following PMSA treatment. Of these, 157 genes were shared (Fig. 1D). Notably, 789 DEGs were specific to OCs and correspond to RANKL-dependent processes affected by PMSA. Gene ontology (GO) analysis revealed that downregulated genes were associated with transcriptional regulation, whereas upregulated DEGs were enriched in inflammatory and immune response pathways in BMMs and OCs. Additionally, upregulated DEGs in BMMs were involved in metabolic processes (Supplementary Fig. 1). Collectively, these findings indicate that PMSA modulates OC differentiation and suppresses key transcriptional mechanisms underlying RANKL-mediated signaling.

### PMSA represses genes associated with protein kinase activity during OC differentiation

**Fig. 2:**
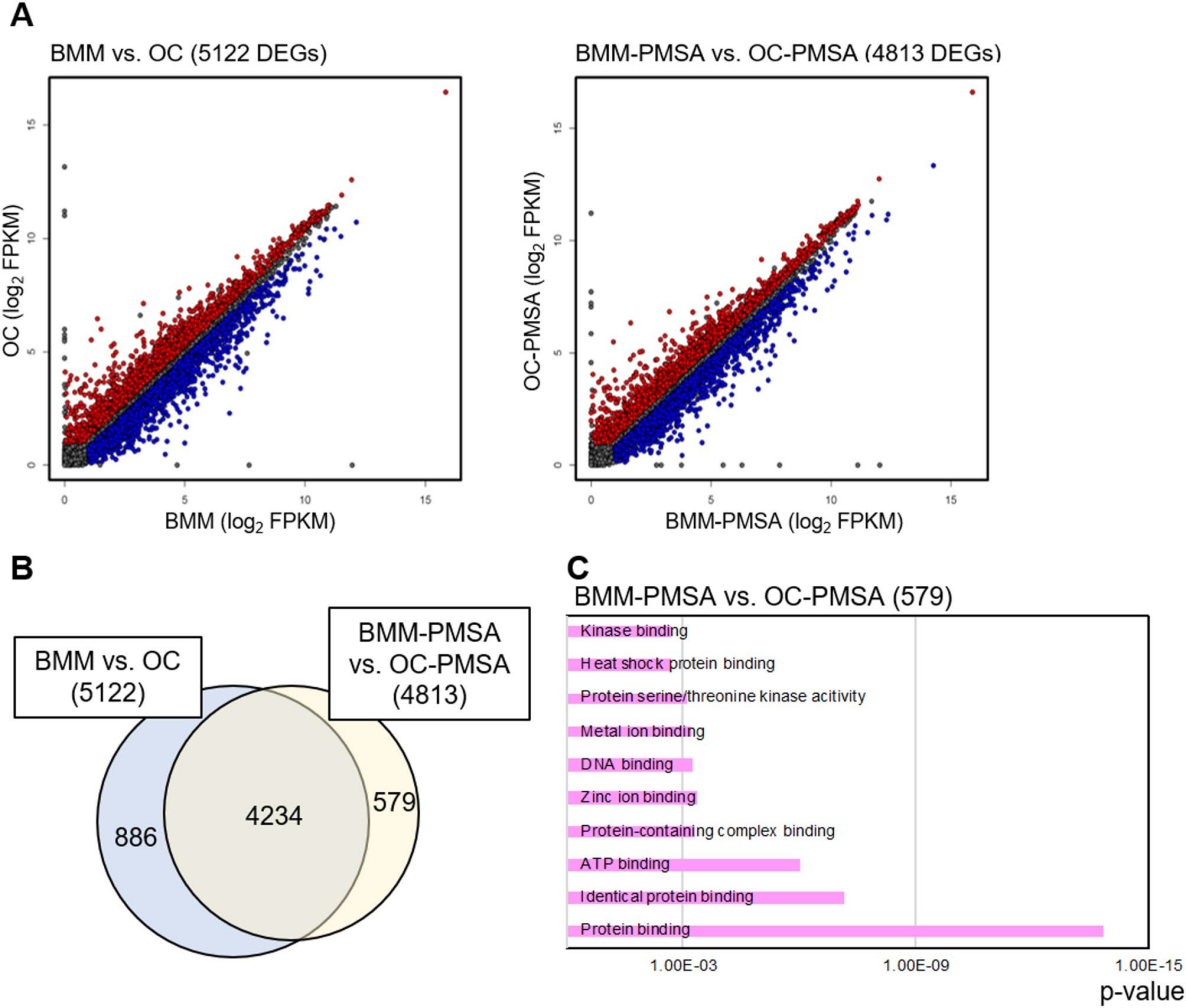
PMSA suppresses genes associated with protein kinase activity during OC differentiation. (**A**) A total of 5122 DEGs were analyzed in the absence of PMSA, and 4813 DEGs were analyzed in PMSA-treated BMMs and OCs. (**B**) Venn diagram showing the overlap of DEGs between PMSA-treated and untreated groups during OC differentiation. (**C**) The 579 PMSA-responsive DEGs were analyzed for molecular function using GO analysis.

To determine whether PMSA regulates distinct pathways during osteoclastogenesis, DEGs were compared in BMMs and OCs with or without PMSA treatment. In the absence of PMSA, RANKL stimulation altered 5122 genes during OC differentiation (Fig. 2A). In PMSA-treated BMMs and OCs, 4813 DEGs were identified. Comparison of these two datasets revealed that 4234 DEGs overlapped between PMSA-treated and untreated conditions (Fig. 2B), indicating that these genes are generally dysregulated during osteoclastogenesis and are unlikely to represent PMSA-specific targets. The remaining 579 DEGs, therefore, constitute putative PMSA-responsive genes.

Of these 579 DEGs, only a small subset overlapped with the PMSA-dependent changes shown in Fig. 1C: 23 (3.97%) DEGs from the BMM vs. BMM-PMSA comparison and 83 (14.3%) DEGs from the OC vs. OC-PMSA comparison. Therefore, most of the 579 DEGs likely reflect PMSA-mediated regulation that occurs during OC differentiation rather than changes restricted to either lineage alone. GO term analysis of the 579 DEGs revealed enrichment for genes related to protein binding (174 DEGs) and protein kinase activity (18 DEGs) (Fig. 2C). Interestingly, most kinase-related genes were downregulated during OC differentiation, suggesting that PMSA suppresses kinase-associated signaling throughout osteoclastogenesis.

As PMSA was previously shown to inhibit OC differentiation by blocking RANKL-induced NFATc1 nuclear translocation, NFATc1-related genes were examined in the RNA-sequencing dataset (Table 1). PMSA reduced *NFATc1* and *NFATc3* RNA expression levels in BMMs and OCs. In addition, protein phosphatase 3 regulatory subunit b, alpha, encoding CaN B, and glycogen synthesis kinase 3 beta were downregulated specifically in OCs. Since CaN B binds calcium and CaM to activate the CaN A catalytic subunit, whether PMSA affected RANKL-induced calcium flux was investigated. PMSA did not impair calcium flux or CaM activity (data not shown), suggesting that RNA expression changes may not directly reflect protein activity. Although PMSA decreased NFATc1 mRNA expression, other components of the NFATc1 signaling cascade were not significantly affected.

**Table 1:** NFATc1-related genes detected by RNA-sequencing.

Gene	BMM vs. OC	BMM-PMSA vs. OC-PMSA	BMM vs. BMM-PMSA	OC vs. OC-PMSA	Description
*Nfatc1*	0.823	0.651	− 0.344	− 0.517	Nuclear factor of activated T cells, cytoplasmic, calcineurin dependent 1
*Nfatc2*	− 0.325	–	-0.367	–	Nuclear factor of activated T cells, cytoplasmic, calcineurin dependent 2
*Nfatc3*	–	–	-0.328	− 0.353	Nuclear factor of activated T cells, cytoplasmic, calcineurin dependent 3
*Ppp3cɑ*	− 0.707	− 0.636	–	–	Protein phosphatase 3, catalytic subunit, alpha isoform
*Ppp3r1*	–	–	–	− 0.300	Protein phosphatase 3, regulatory subunit B, alpha isoform (calcineurin B, type I)
*Rcan1*	0.464	0.342	–	–	Regulator of calcineurin 1
*Gsk3β*	− 0.535	− 0.591	–	− 0.279	Glycogen synthase kinase 3 beta
*Csnk1α1*	0.417	0.400	–	–	Casein kinase 1, alpha 1
*Cabin1*	0.530	0.473	–	–	Calcineurin binding protein 1
*Stim1*	− 0.839	− 0.592	–	–	Stromal interaction molecule 1
*Orai1*	− 0.382	–	–	–	ORAI calcium release-activated calcium modulator 1

The numbers indicate log_2_ fold change values that are statistically significant, q-value < 0.05.

### NFATc1 interacts with CaN in the presence of PMSA

**Fig. 3:**
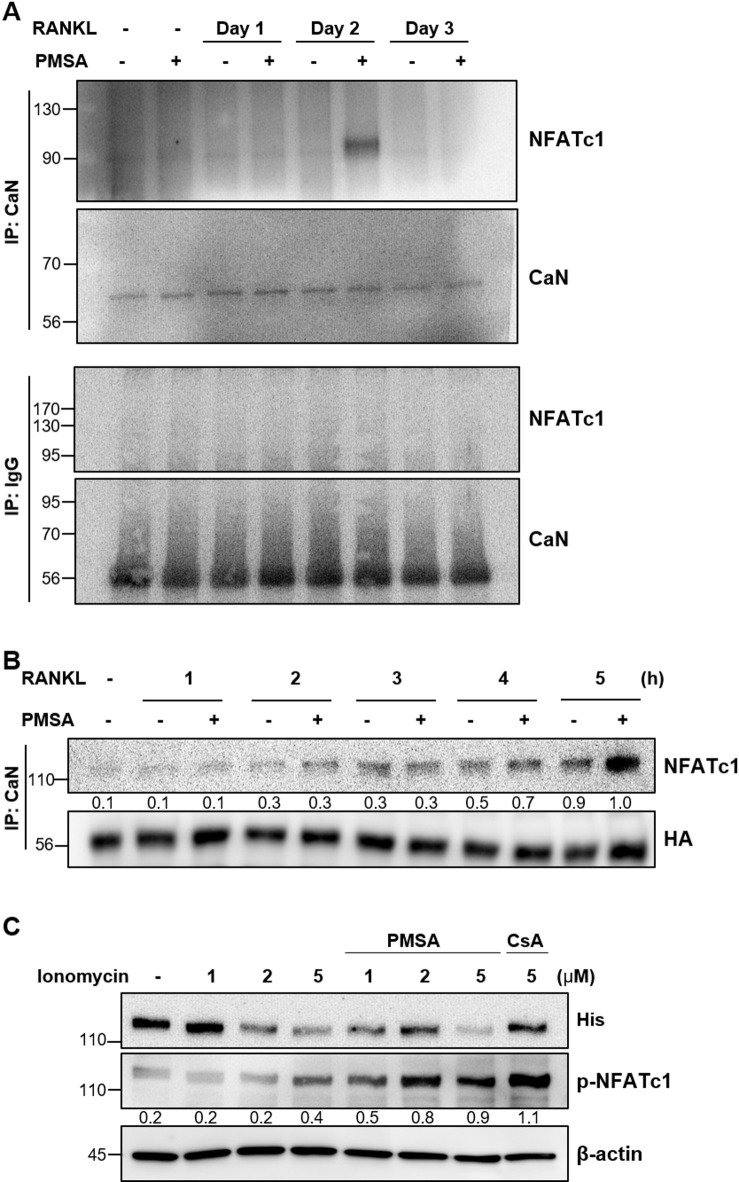
NFATc1 interacts with CaN in the presence of PMSA. (**A**) Immunoprecipitation (IP) analysis of BMMs treated with RANKL and 10 µM PMSA for the indicated durations. IgG was used as a negative control. (**B**) Immunoprecipitation of RAW264.7 cells overexpressing HA-tagged CaN following RANKL stimulation. (**C**) Immunoprecipitation of 293 T cells overexpressing His-tagged NFATc1 following ionomycin stimulation. Ionomycin concentrations are indicated; cells were co-treated with 10 µM PMSA or 2 µM CsA. Phospho (p)-NFATc1 levels were normalized to β-actin.

CaN is a key regulator of NFATc1 activation during osteoclastogenesis^13^. Given this regulatory relationship, it was examined whether PMSA influences the interaction between CaN and NFATc1. BMMs were differentiated into OCs by RANKL stimulation in the presence of PMSA, and NFATc1–CaN binding was assessed by immunoprecipitation. A clear interaction was detected under PMSA-treated conditions at Day 2 after RANKL stimulation, whereas no detectable interaction was observed in untreated controls (Fig. 3A). These findings suggest that PMSA promotes a strong interaction between NFATc1 and CaN.

To further validate this hypothesis, RAW264.7 cells were transfected with hemagglutinin (HA)-tagged CaN and subjected to co-immunoprecipitation following RANKL stimulation (Fig. 3B). Consistent with our initial observations, NFATc1 strongly co-precipitated with HA-tagged CaN within 4‒5 h of RANKL stimulation, with the interaction markedly enhanced in the presence of PMSA. In contrast, only minimal interaction was observed without PMSA. Additionally, NFATc1-overexpressing 293 T cells exhibited elevated phospho-NFATc1 levels after co-treatment with ionomycin and PMSA, comparable to the increase observed with CsA treatment (Fig. 3C). As ionomycin induces a strong calcium influx and inhibits calcium channel endocytosis^22^, the increase in phospho-NFATc1 levels likely reflects impaired CaN-mediated dephosphorylation despite enhanced CaN–NFATc1 association. These findings suggest that although PMSA stabilizes the CaN‒NFATc1 interaction, it may simultaneously hinder CaN catalytic function, resulting in increased phospho-NFATc1 levels.

Interestingly, comparison of binding kinetics between RAW264.7 cells and BMMs revealed distinct temporal patterns—hours versus days, respectively. This disparity may arise from differences in cellular composition: BMMs comprise multiple hematopoietic lineages, whereas RAW264.7 cells are a relatively homogeneous monocytic cell line. Besides, M-CSF is required to maintain BMMs for cell survival and differentiation, whereas RAW264.7 cells can differentiate with RANKL stimulation alone^23^ . Variations in receptor activator of nuclear factor kappa B (RANK) expression levels or signaling responsiveness between these models may also contribute to the differential timing observed during OC differentiation. Additionally, proteomic analyses revealed differences between BMMs and RAW264.7 cells, including constitutive Erk and Akt activation in RAW264.7 cells and distinct NFATc1 expression patterns during osteoclast differentiation^23^.

### PMSA inhibits the phosphatase activity of CaN

**Fig. 4:**
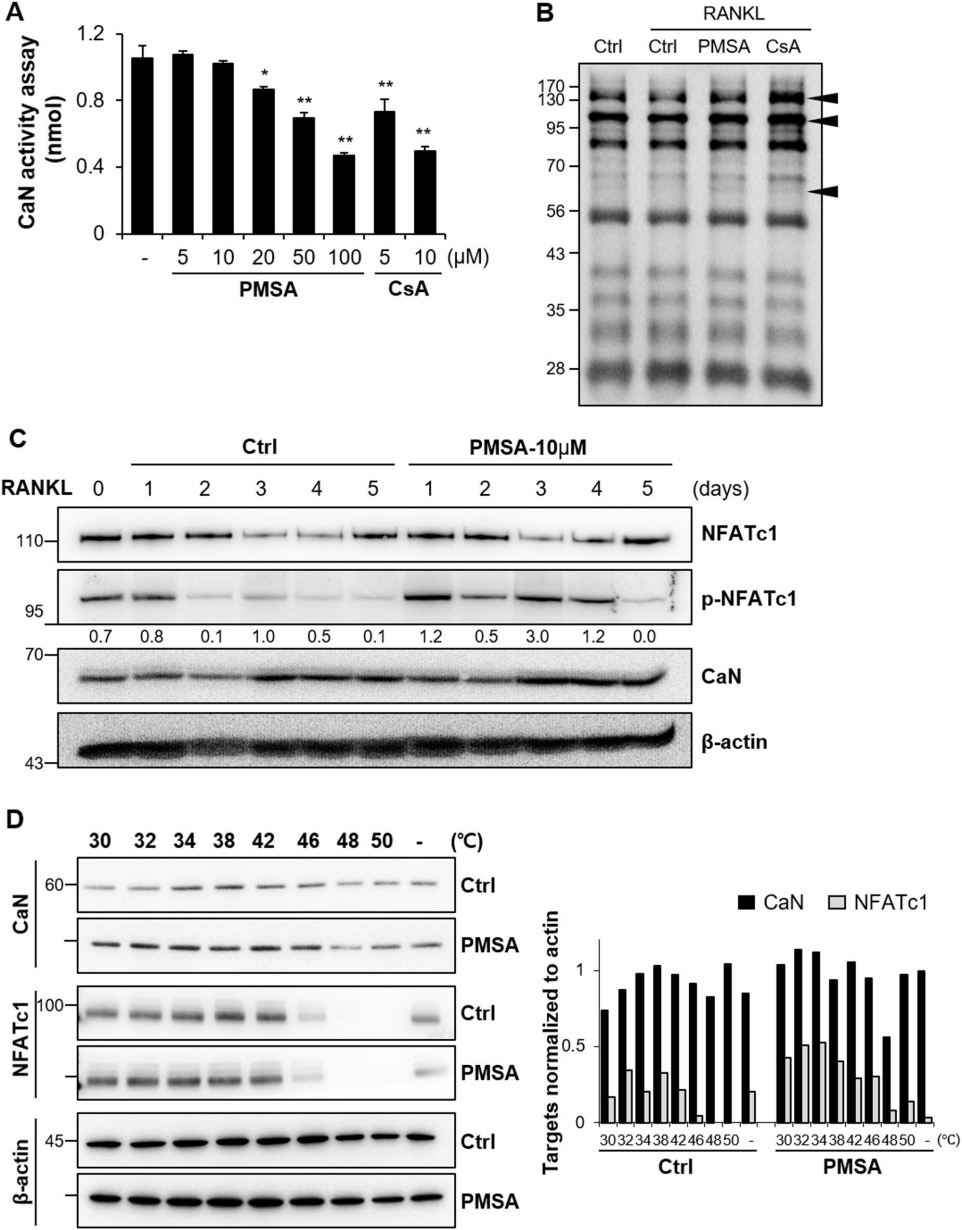
PMSA inhibits the phosphatase activity of CaN. (**A**) CaN activity was measured in the presence of PMSA or CsA. Data represent the mean ± standard deviation of three replicates. *, *P* < 0.05; **, *P* < 0.01 (**B**) The immunoprecipitated samples were immunoblotted with antibodies against phosphorylated serine/threonine residues.  Arrowheads indicate proteins with increased immunoreactivity. Cells were treated with PMSA or CsA for 24 h and stimulated with RANKL for 5 h. (**C**) Western blotting of RAW264.7 cells following RANKL stimulation. P-NFATc1 levels were normalized to β-actin and total NFATc1 levels. (**D**) Thermal shift assay of RAW264.7 cells treated with PMSA. Cell lysates were exposed to temperatures ranging from 30℃ to 50℃. The final sample was not incubated and served as a negative control (Ctrl). The graph shows CaN and NFATc1 expression levels normalized to β-actin. All Western blots were repeated at least twice.

To investigate whether PMSA regulates the enzymatic activity of CaN, a dephosphorylation assay was performed. As shown in Fig. 4A, PMSA inhibited CaN phosphatase activity in a dose-dependent manner, closely resembling the inhibitory effects observed with CsA. To further evaluate phosphatase activity, immunoblotting was performed using antibodies specific for phosphorylated serine and threonine residues in immunoprecipitated samples. RAW264.7 cells overexpressing HA-tagged CaN were co-treated with RANKL and either PMSA or CsA, followed by immunoprecipitation with anti-HA antibodies. PMSA treatment produced a modest increase in several protein bands, whereas CsA treatment resulted in a more pronounced accumulation of these bands (arrowheads in Fig. 4B).

Our previous findings identified NFATc1 as a potential PMSA target^20^. Although total NFATc1 expression remained unchanged during OC differentiation, PMSA treatment markedly increased phospho-NFATc1 in OCs (Fig. 4C), while CaN protein levels remained unchanged. This PMSA-mediated regulation of NFATc1 was further validated by two-dimensional gel immunoblotting of the immunoprecipitated proteins, which revealed distinct spots corresponding to the expected size and isoelectric point of NFATc1 (star in Supplementary Fig. 2).

To determine whether CaN directly interacts with PMSA, a thermal shift assay was performed in RAW264.7 cells. Lysate from PMSA-treated cells exhibited a strong CaN band at 30ー32℃, whereas control lysates displayed only a faint band at these temperatures (Fig. 4D). In contrast, NFATc1 expression remained unchanged following PMSA treatment. These results indicate that PMSA directly binds to CaN and stabilizes it under conditions that typically promote protein destabilization. Collectively, these findings demonstrate that PMSA directly binds to CaN and inhibits its phosphatase activity toward target proteins such as NFATc1.

## Discussion

It has been demonstrated that PMSA inhibits dephosphorylation of NFATc1 during osteoclastogenesis^20^. To further elucidate the mechanism of PMSA, RNA-sequencing and proteomic analyses were performed in this study. RNA-sequencing data revealed PMSA involvement in oxidative process, immune response, and transcriptional regulation, with corresponding up- and down-regulated genes (Supplementary Fig. 1). To identify osteoclast-specific targets, we compared dysregulated genes during osteoclast differentiation. PMSA treatment altered genes related to protein binding and kinase activities following RANKL stimulation (Fig. 2C). As a regulator of NFATc1 representing in our previous report, we examined CaN, the phosphatase that dephosphorylates NFATc1. Although CaN expression levels were unchanged in our transcriptional and proteomic data, these findings suggest PMSA modulates CaN enzymatic activity.

Based on RNA-sequencing results, CaN may not be the only kinase regulated by PMSA; additional kinases, including mitogen-activated protein kinases (MAPKs), glycogen synthase kinases (GSKs), and cyclin-dependent kinases (CDKs), may also be affected. When the DEGs shown in Fig. 2C were analyzed, MAPKs, calmodulin-dependent protein kinases 2 gamma (CaMK2γ), inhibitor of nuclear factor kappa B kinase subunit epsilon (IKBKε), and GSK-3 were identified in protein serine and threonine kinase activity by PMSA in osteoclasts. In comparison with the increased binding of phosphorylated proteins to CaN (Fig. 4B), these kinases may represent putative targets of PMSA. However, these DEGs were downregulated by PMSA in osteoclasts following 24 h of stimulation, whereas hyperphosphorylated proteins were affected by acute RANKL stimulation. Dysregulation of calcineurin has been implicated in various severe diseases through the hyperphosphorylation of its substrate kinases^11,24^. NFATs and MAPKs are disrupted by CaN in cardiac hypertrophy and ovarian cancer^25–27^. Moreover, the interaction between CaN and GSK-3β has been reported in T-cell acute lymphoblastic leukemia^28^. Phosphorylated CaMK2 is dephosphorylated by CaN and is involved in cardiac hypertrophy, Alzheimer’s disease, and muscle dysfunction^29,30^. It is possible that the activity of MAPKs and CaMKs is regulated by PMSA. NFATs regulate several genes involved in cell cycle control during cell differentiation^31^. Although PMSA may not directly regulate all of these kinases, our data indicate that it acts as a potential regulator of CaN activity, within the limitations of our study.

Calcium-mediated signaling is essential for osteoclastogenesis^32^. The RANKL–RANK interaction activates downstream phospholipase C gamma, increasing intracellular calcium concentrations and promoting CaN activation^33^. This cascade leads to NFATc1 dephosphorylation, enabling transcription of key OC differentiation genes, including tartrate-resistant acid phosphatase, cathepsin K, and OC-associated receptor, within the nucleus^34^. Activated CaN is cleaved by the calcium-dependent cysteine protease calpain, generating a truncated form lacking the autoinhibitory domain and localizing to the nucleus^35^. Such changes in subcellular localization modify substrate accessibility and dephosphorylation dynamics^36^. CaN activity is typically assessed by monitoring NFAT phosphorylation or transcriptional activity. In this study, CaN function was quantified using a synthetic substrate and phosphorylation levels of NFATc1 (Fig. 4). The CaN assay system contained the catalytic CaN A subunit and regulatory CaN B subunit as a heterodimer. PMSA inhibited the enzymatic activity of CaN without disrupting complex formation. Other approaches, such as NFAT reporter assays or nuclear localization, are available; however, NFAT reporter assays cannot distinguish among NFAT isoforms, and immunoassays cannot selectively detect the cleaved form of CaN.

CaN inhibitors also exhibit anti-cancer properties. CsA induces apoptosis in lymphoma and leukemia cell lines^37^. Additionally, CsA and tacrolimus suppress proliferation and migration in bladder, prostate^38,39^, and breast cancer cells^40^. In our previous viability assays, BMMs remained viable at an effective PMSA concentration, although higher doses (e.g., 100 µM) reduced cell viability (data not shown). Although PMSA requires a higher inhibitory concentration for CaN than CsA, our findings suggest that PMSA could potentially serve as an immunosuppressant.

**Fig. 5:**
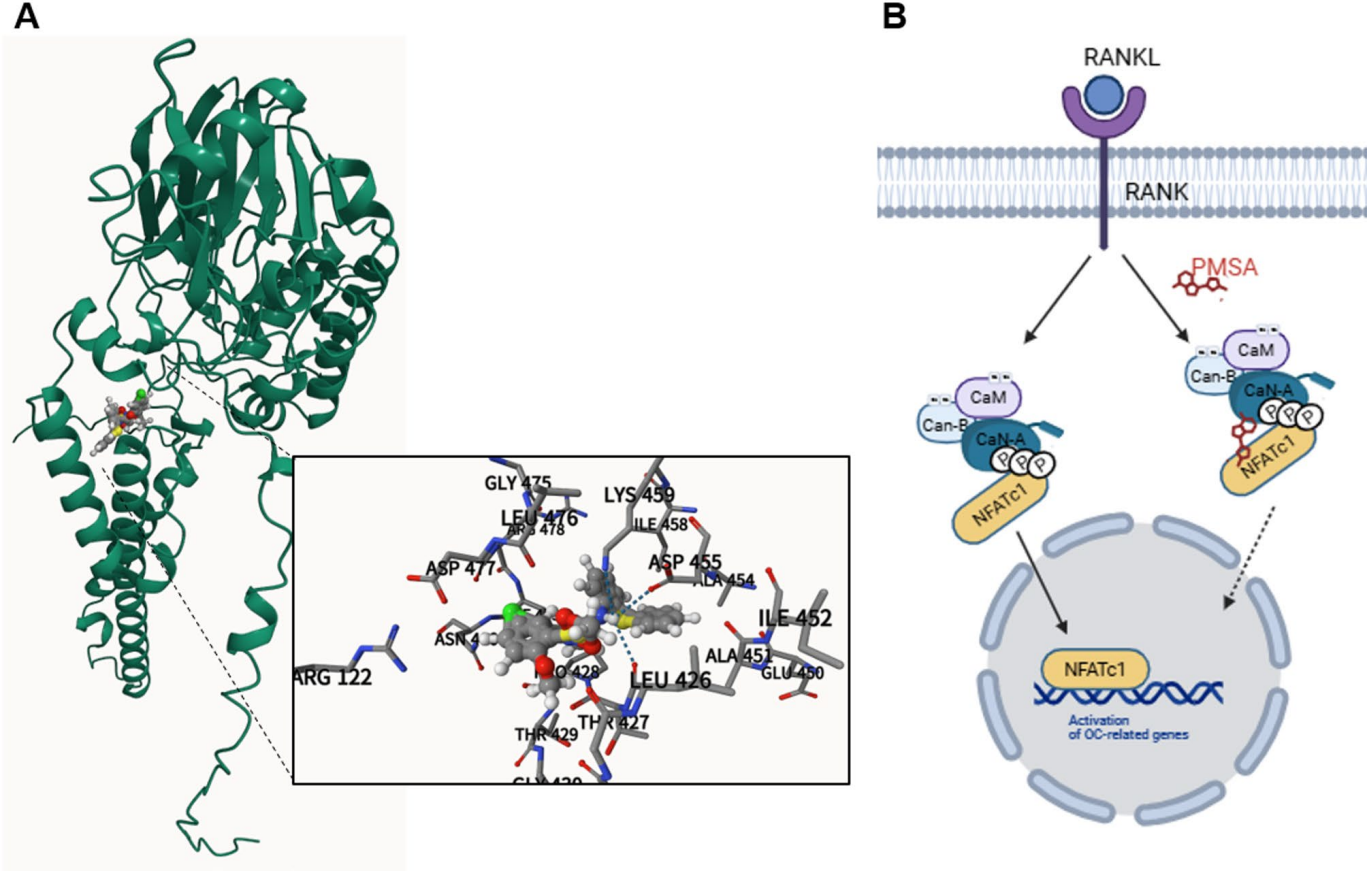
Working model of PMSA modulation of CaN-NFATc1 signaling. (**A**) In silico docking analysis was performed with AD3 platform v.4.9.0 (Arontier Inc., https://ad3.io/). CaN protein is shown as a green ribbon structure; PMSA is depicted as a ball-and-stick model (gray = C, red = O, green = Cl). (**B**) Schematic illustrating PMSA binding to CaN, stabilizing the CaN-NFATc1 interaction while inhibiting dephosphorylation and osteoclast differentiation. All schematic illustrations were created in BioRender (https://BioRender.com/).

CsA and tacrolimus bind to the conserved LxVP consensus motif of CaN^11^. To assess whether PMSA binds to the LxVP site like other immunosuppressants, we performed in silico docking analysis (Fig. 5A). Although further validation is needed, PMSA appears to bind near the LxVP substrate binding region^11,41^. In the presence of calcium and CaM, immunosuppressant complexes occlude the LxVP pocket, thereby preventing substrate access. Based on our findings, it is proposed that PMSA occupies this site and promotes a stable interaction between CaN and NFATc1, reinforcing complex formation rather than facilitating substrate release.

Collectively, our data support the conclusion that PMSA acts as a potent inhibitor of osteoclastogenesis and bone resorption. Moreover, PMSA acts as a putative modulator of the CaN-NFATc1 signaling pathway, suppressing NFATc1 dephosphorylation while stabilizing their interaction between CaN and NFATc1 (Fig. 5B). These properties highlight PMSA as a potential immunosuppressive agent with therapeutic relevance for transplant recipients.

## Materials and methods

### RNA-sequencing

Bone marrow derived macrophages (BMMs) were isolated from the femurs and tibias of mice a described previously^20^; all animal procedures were approved by the IACUC at Chonnam National University (CNU IACUC-YB-2023–19) and performed in accordance with national guidelines for the care and use of laboratory animals. BMMs cultured in 30 ng/mL M-CSF containing aMEM medium (Thermo Fisher Scientific, USA). For RNA-sequencing, BMMs were incubated with 30 ng/mL M-CSF and/or 50 ng/mL RANKL (Thermo Fisher Scientific) for 24 h in the presence of 10 µM PMSA, a concentration previously identified as strongly inhibitory to osteoclastogenesis^20^. Total RNAs were extracted using the RNeasy kit (Qiagen, Germany) following the manufacturer’s instructions.

Total RNA concentration was measured using the Quant-IT RiboGreen assay (Thermo Fisher Scientific), and RNA integrity was assessed with the TapeStation RNA ScreenTape system (Agilent Technologies, USA). Only samples with RNA integrity number > 7.0 were used for library construction.

RNA libraries were prepared from 0.5 µg of total RNA per sample using the Illumina TruSeq Stranded Total RNA Library Prep Gold Kit (Illumina Inc., USA). Ribosomal RNA (rRNA) was removed using the Ribo-Zero rRNA Removal Kit (Human/Mouse/Rat Gold) (Illumina Inc.). The remaining mRNA was fragmented using divalent cations at elevated temperature and reverse-transcribed using with SuperScript II reverse transcriptase (Thermo Fisher Scientific) and random primers to generate first-strand complement deoxyribonucleic acid (cDNA).

Second-strand cDNA synthesis was performed using DNA polymerase I, RNase H, and deoxyuridine triphosphate. The resulting cDNA underwent end repair, ‘A-tailing, and adaptor ligation, followed by polymerase chain reaction (PCR) enrichment to create the final libraries.

Library quantification was performed using KAPA Library Quantification kits for Illumina platforms (KAPA Biosystems, USA), and library quality was confirmed with the TapeStation D1000 ScreenTape system (Agilent Technologies). Indexed libraries were sequenced on an Illumina NovaSeq platform (paired-end 2 × 100 base pairs; Illumina Inc.) by Macrogen Inc.

The raw sequence reads were processed as previously described^42^. DEGs were identified using the criteria: fragments per kilobase of exon per million fragments mapped > 1 in at least one sample and q-value < 0.05. Gene enrichment analysis was performed using GO categories using the DAVID functional annotation tool (https://www.david.ncifcrf.gov). The mouse reference genome and annotation files were obtained from the University of California, Santa Cruz Genome Browser (https://www.genome.ucsc.edu). R software was used for data visualization.

### Immunoprecipitation

Immunoprecipitation was performed as previously described^43^. Briefly, RAW264.7 cells treated with RANKL and/or PMSA were lysed and incubated with agarose beads conjugated with protein A/G (Santa Cruz Biotechnology, USA) and either rabbit immunoglobulin G (Santa Cruz Biotechnology), CaN (Cell Signaling Technology, CST, USA), or HA (Sigma-Aldrich, USA) antibodies. After three washes with phosphate-buffered saline, the immunoprecipitated proteins were eluted and subjected to Western blotting using NFATc1 or phospho-serine/threonine antibodies (Cell Signaling Technology).

### Two-dimensional Western blotting

The immunoprecipitated proteins from CaN-HA–overexpressing RAW264.7 cells (pCMV-HA plasmid containing the subcloned human CaN gene) incubated with PMSA for 24 h were resuspended in immobilized pH gradient (IPG) buffer (pH 3–10 NL; Cytiva, USA) and applied to 7 cm IPG strips pH 3-10NL (Cytiva).

Proteins were separated at 8,000 V for 20,000 Vh using the Ettan™ IPGphore II™ system (GE Healthcare, USA). Before sodium dodecyl sulfate (SDS)-polyacrylamide gel electrophoresis (PAGE), IPG strips were sequentially equilibrated for 15 min each in SDS equilibration buffers containing 6 M urea, 2% SDS, 0.075 M TRIS-hydrochloric acid (pH 8.8), 30% glycerol, and 2% tributylphosphine, supplemented with 100 mg dithiothreitol for the first step and 250 mg iodoacetamide for the second. Western blotting was performed, followed by protein separation on 8% SDS-PAGE gels.

The following primary antibodies were used: NFATc1 (#8032) and CaN (#2614) (Cell Signaling Technology, Danvers, MA, USA); HA (Santa Cruz Biotech., sc-7392); phospho-NFATc1 (Invitrogen, PA5-64696); and β-actin (Sigma-Aldrich). Horseradish peroxidase-conjugated anti-rabbit and anti-mouse secondary antibodies were procured from Cell Signaling Technology. All antibodies were used at a 1:1000 dilution in 5% skim milk, as recommended by the manufacturers.

### Cellular thermal shift assay

RAW264.7 cells were treated with 50 µM PMSA for 3 h under standard culture conditions, then harvested by centrifugation. Aliquots of the cells were subjected to a temperature gradient (30, 32, 34, 38, 42, 46, 48, and 50 °C) for 5 min using a PCR thermal block instrument (Bio-Rad Laboratories Inc., USA). Following thermal stimulation, cells were lysed in radioimmunoprecipitation assay buffer (Thermo Fisher Scientific) supplemented with protease and phosphatase inhibitors. Lysates were clarified by centrifugation, denatured with loading buffer, and boiled. Protein expression levels were subsequently analyzed by Western blotting.

### CaN phosphatase activity assay

CaN activity was measured using a CaN assay kit (Enzo Life Sciences, USA), following the manufacturer’s instructions. Briefly, PMSA or CsA was incubated with regulatory subunit type II substrate, followed by the addition of a mixture containing CaN and CaM. After 30 min, the reactions were terminated, and the absorbance was measured at 620 nm.

### Statistical analysis

All data are presented as the mean ± standard deviation from three independent experiments, unless otherwise specified. Statistical significance between the control and PMSA-treated groups was determined using unpaired, two-tailed Student’s t-tests.

## Supplementary Information

Below is the link to the electronic supplementary material.

Supplementary Material 1

Supplementary Material 2

## Data Availability

The datasets generated and analyzed during the current study are available at the Gene Expression Omnibus site of the NCBI; PRJNA1396781 https://dataview.ncbi.nlm.nih.gov/object/PRJNA1396781?reviewer=if3f02ghqv4hia1dh6q5f1g76h
