# Supplementary material for: PMSA as a potential modulator of calcineurin phosphatase activity
Source: Sci Rep. 2026 Apr 22;16:18795. doi: 10.1038/s41598-026-48882-9 (PMC13272792; doi:10.1038/s41598-026-48882-9)
Supplement: Supplementary file 1 — Supplementary Material 1 [file 41598_2026_48882_MOESM1_ESM.pdf]

PMSA as a potential modulator of calcineurin phosphatase activity

Supplementary figures

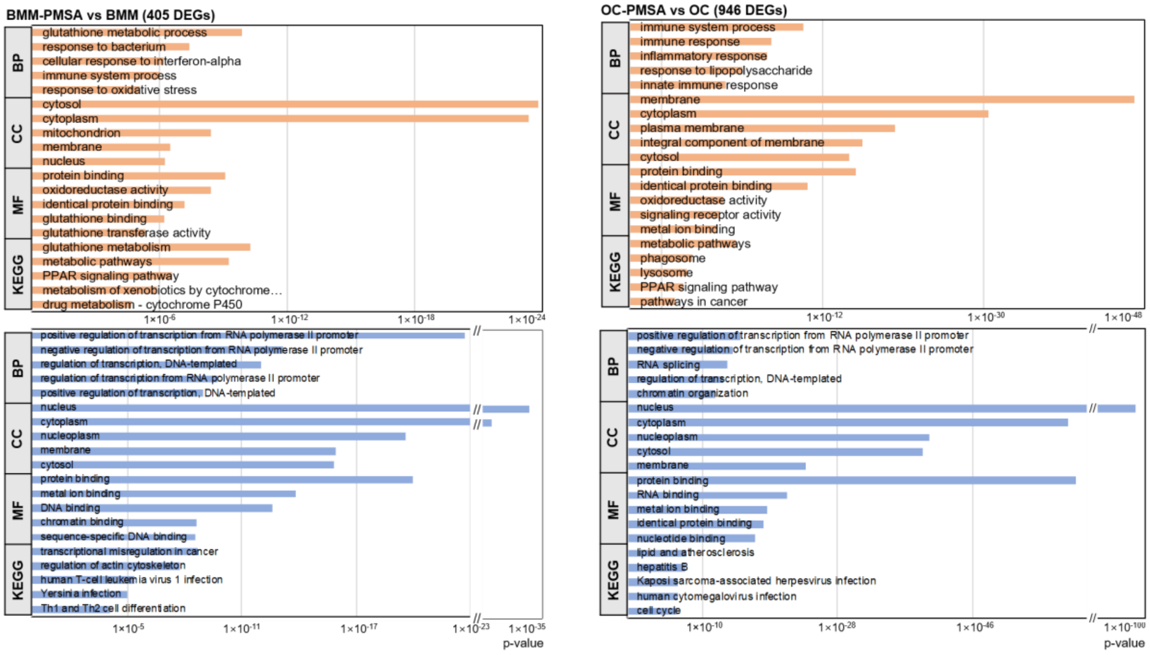

Supplementary Fig. 1. Gene Ontology analysis of 405 and 946 DEGs shown in Fig. 1C.

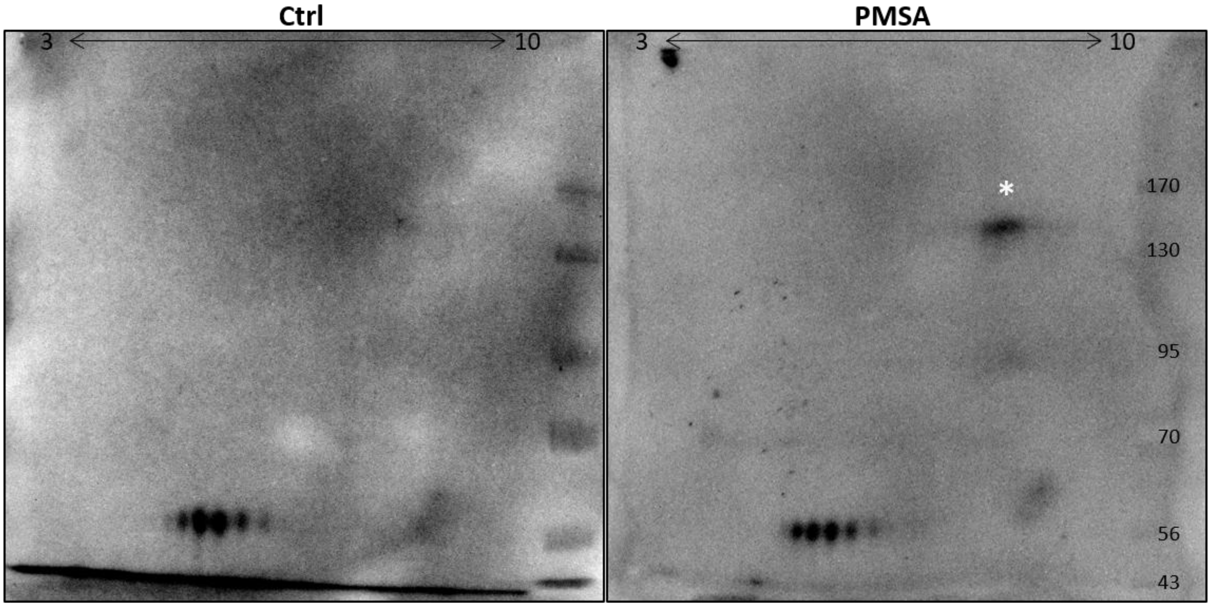

Supplementary Fig. 2. Two-dimensional immunoblotting was performed using NFATc1 antibodies. Cell lysates were precipitated with hemagglutinin (HA) antibodies from RAW264.7 cells overexpressing HA-tagged CaN.
