# Supplementary material for: PMSA as a potential modulator of calcineurin phosphatase activity
Source: Sci Rep. 2026 Apr 22;16:18795. doi: 10.1038/s41598-026-48882-9 (PMC13272792; doi:10.1038/s41598-026-48882-9)

Figure 3-A

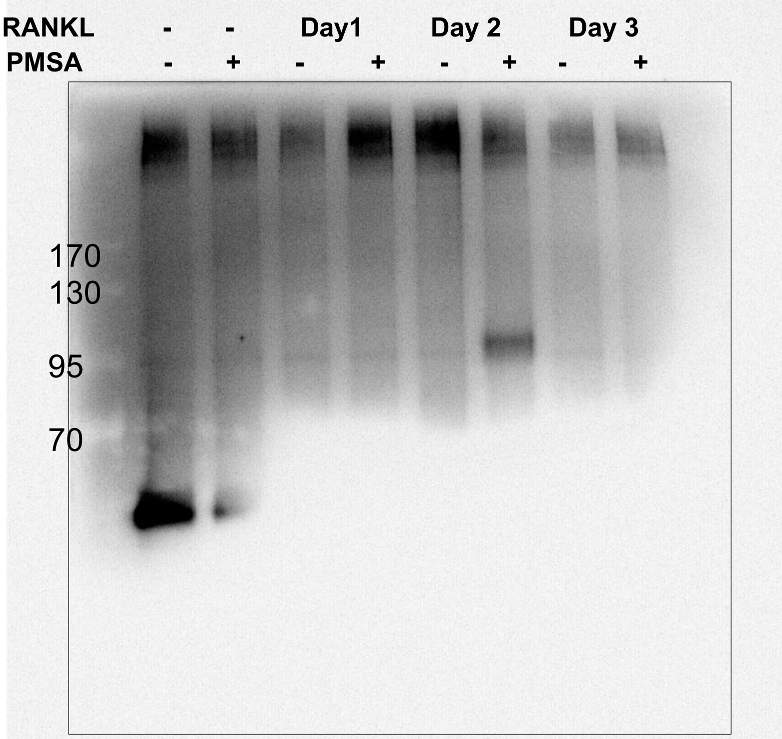

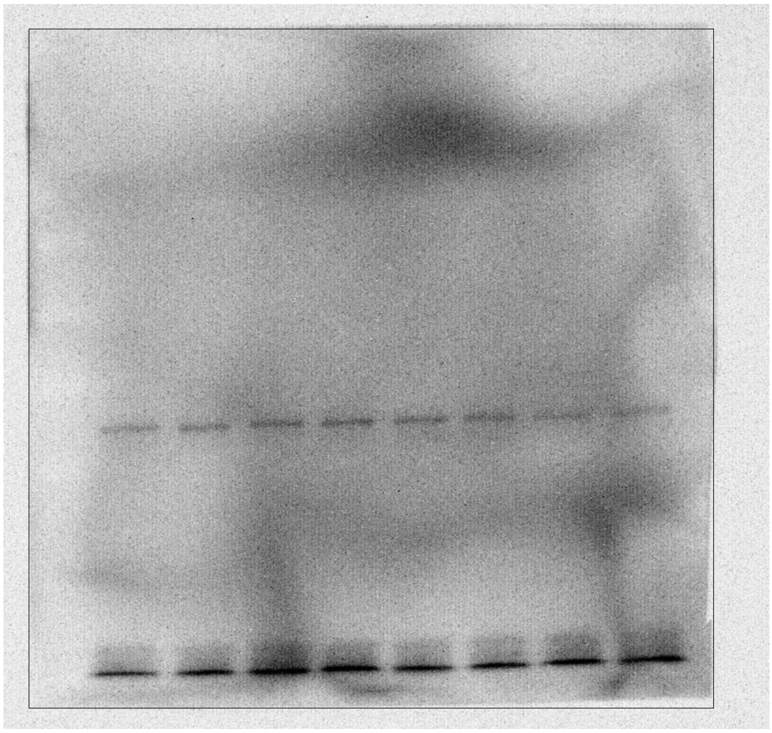

Figure 3-A

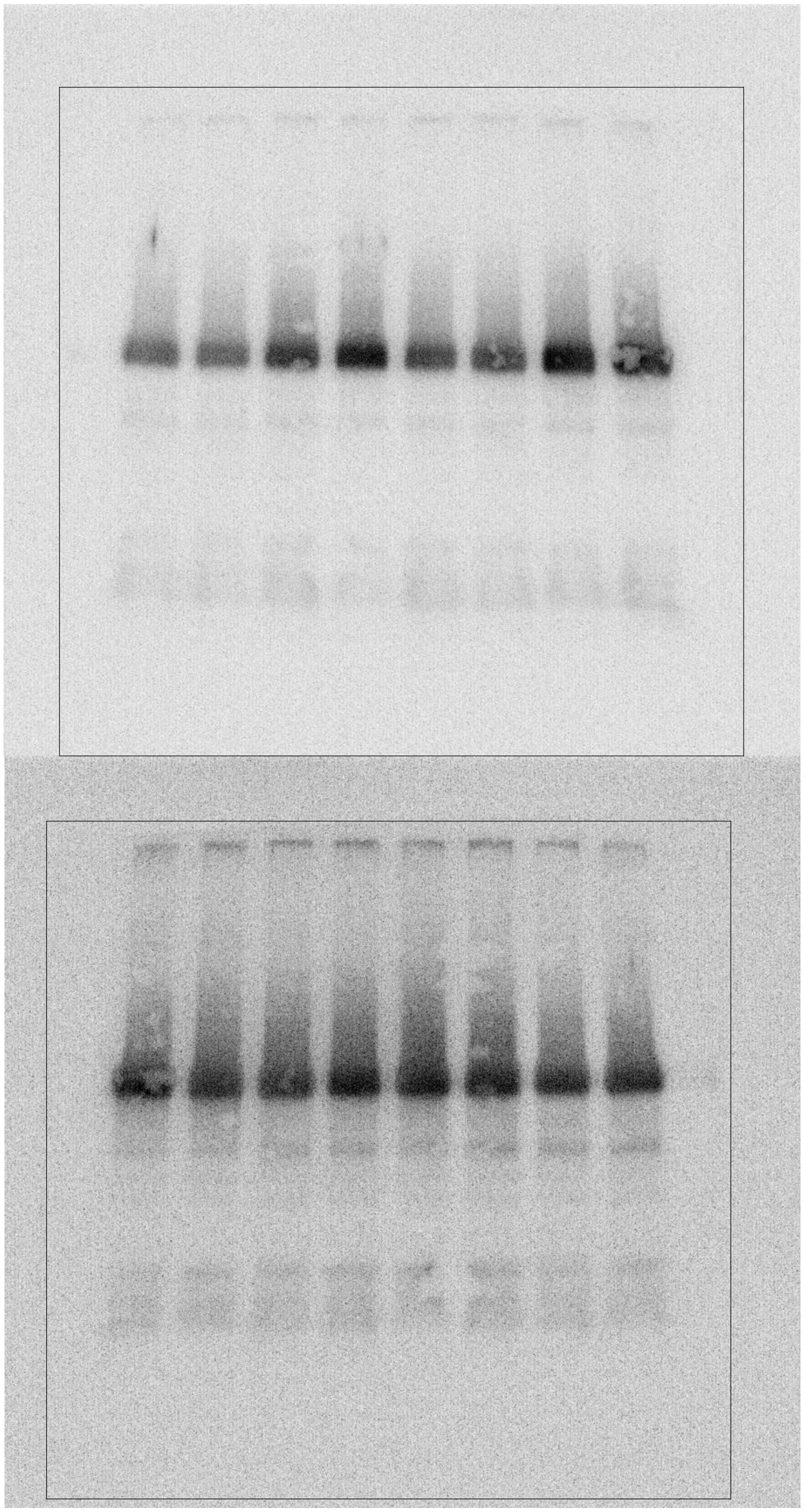

Figure 3-B

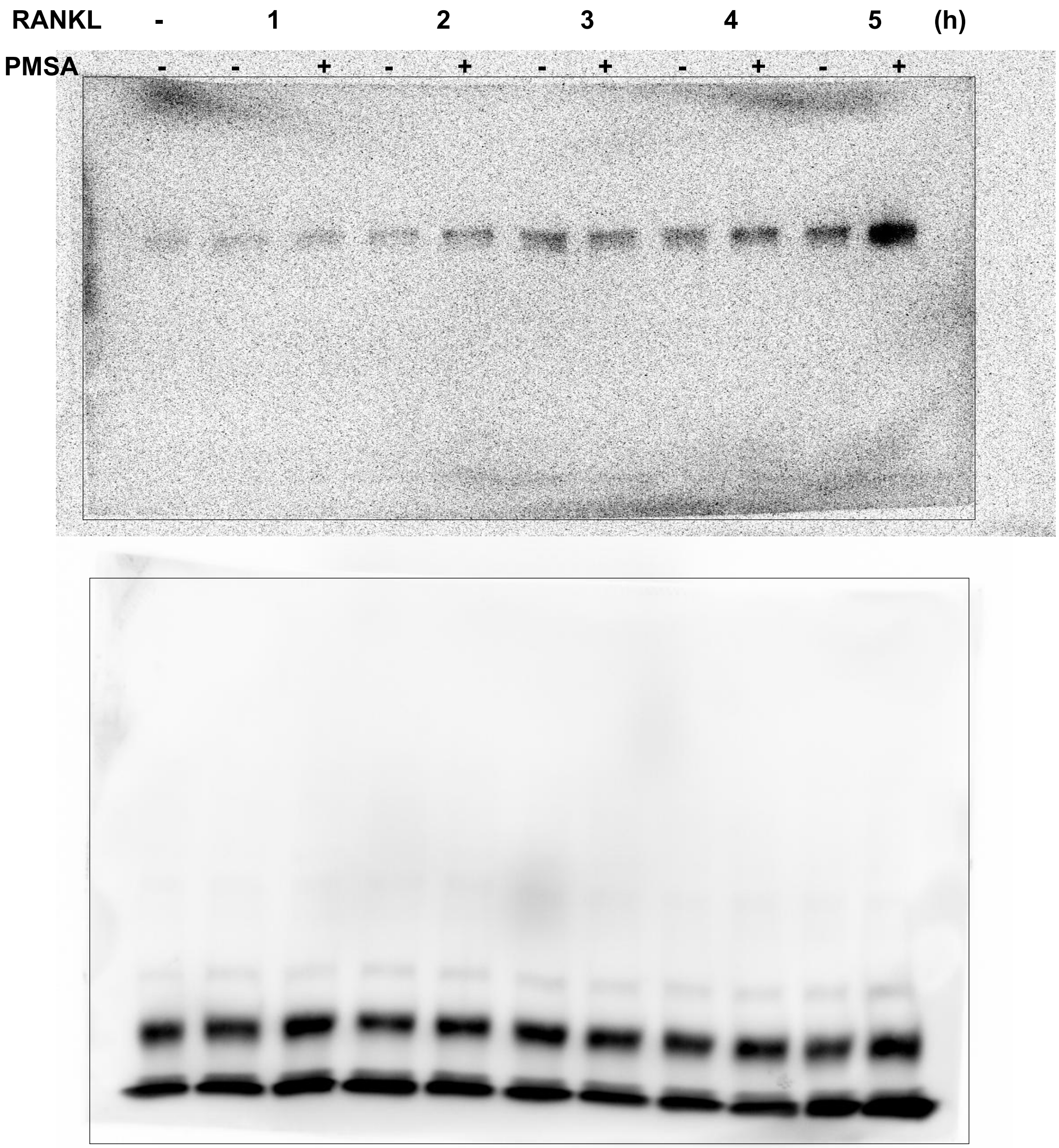

Figure 3-C

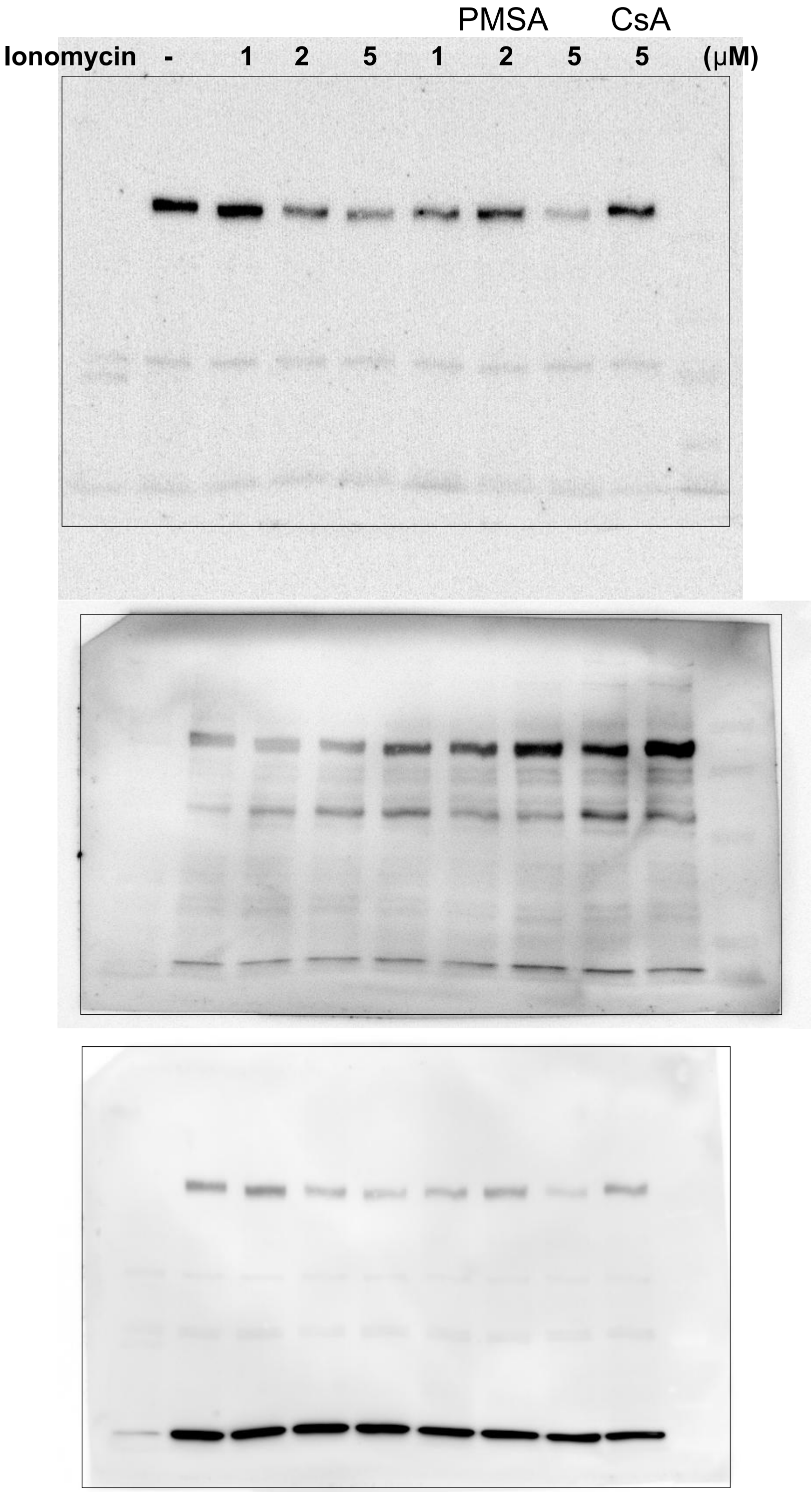

Figure 4-B

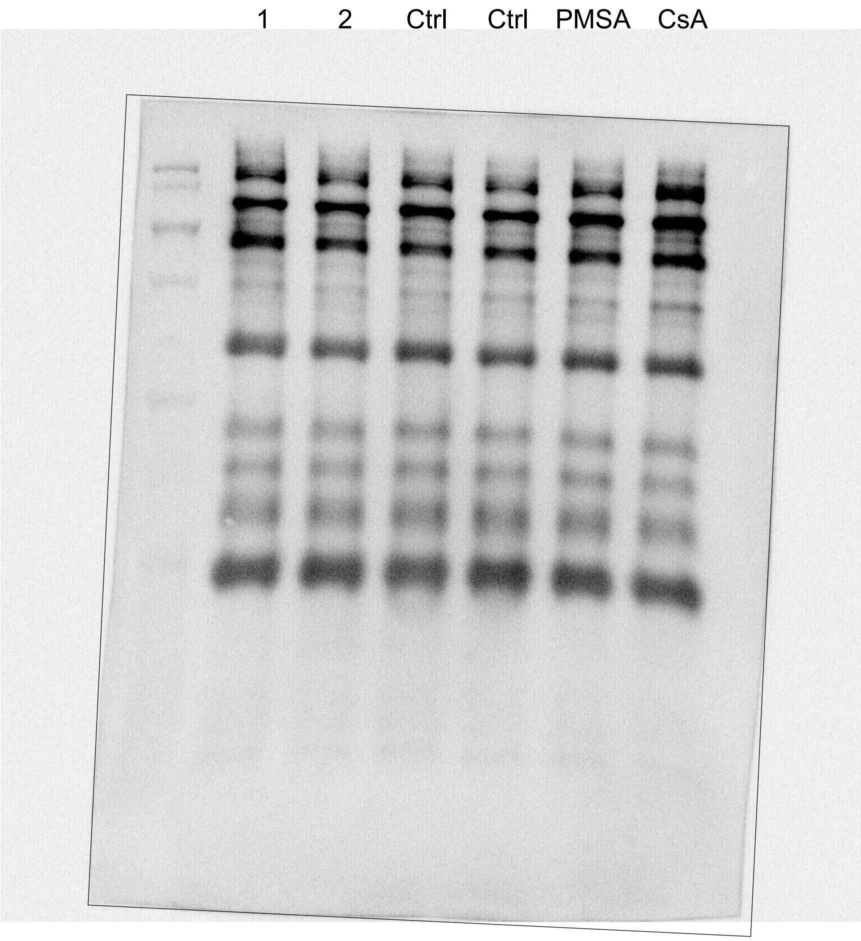

Figure 4-C

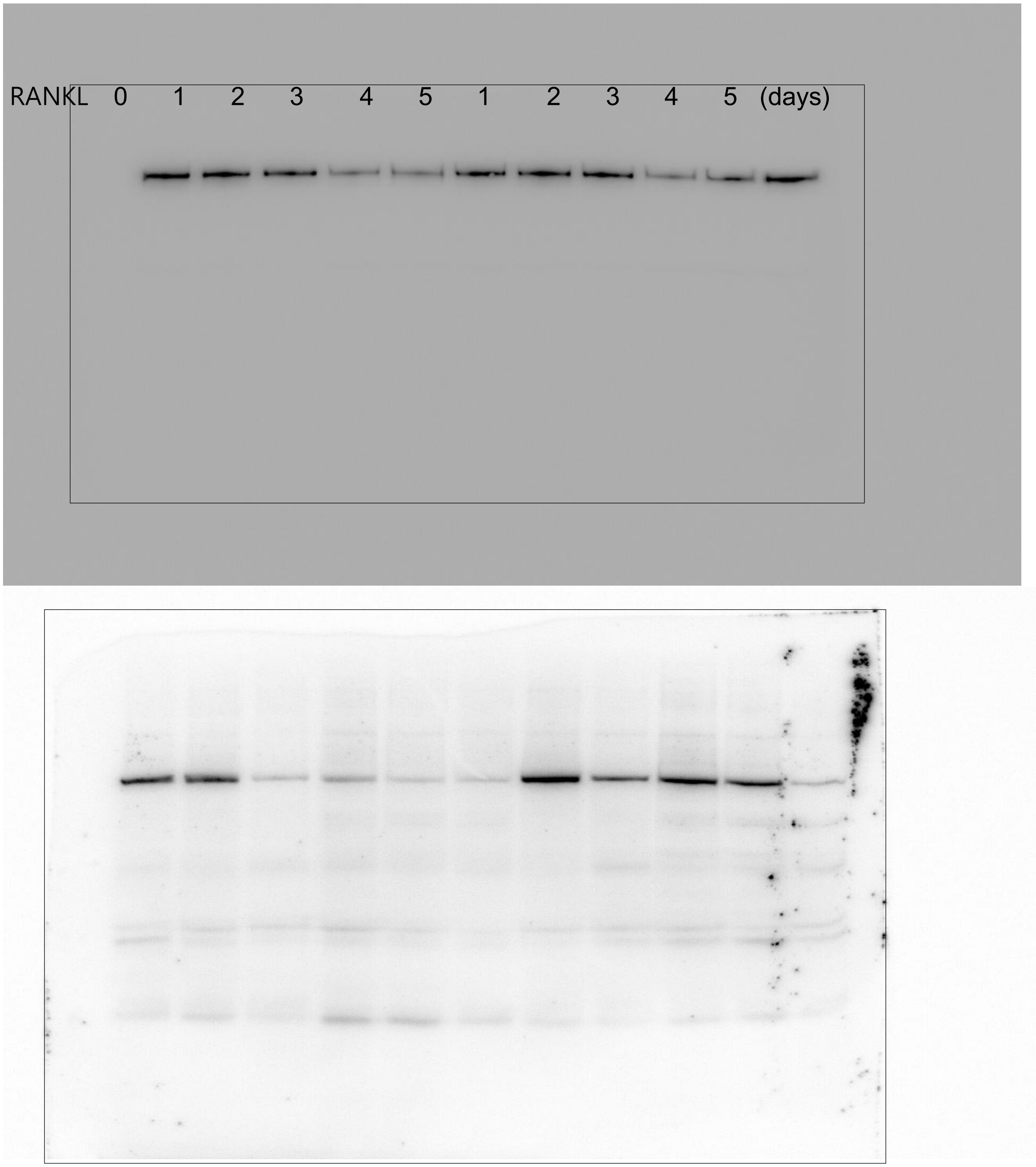

Figure 4C

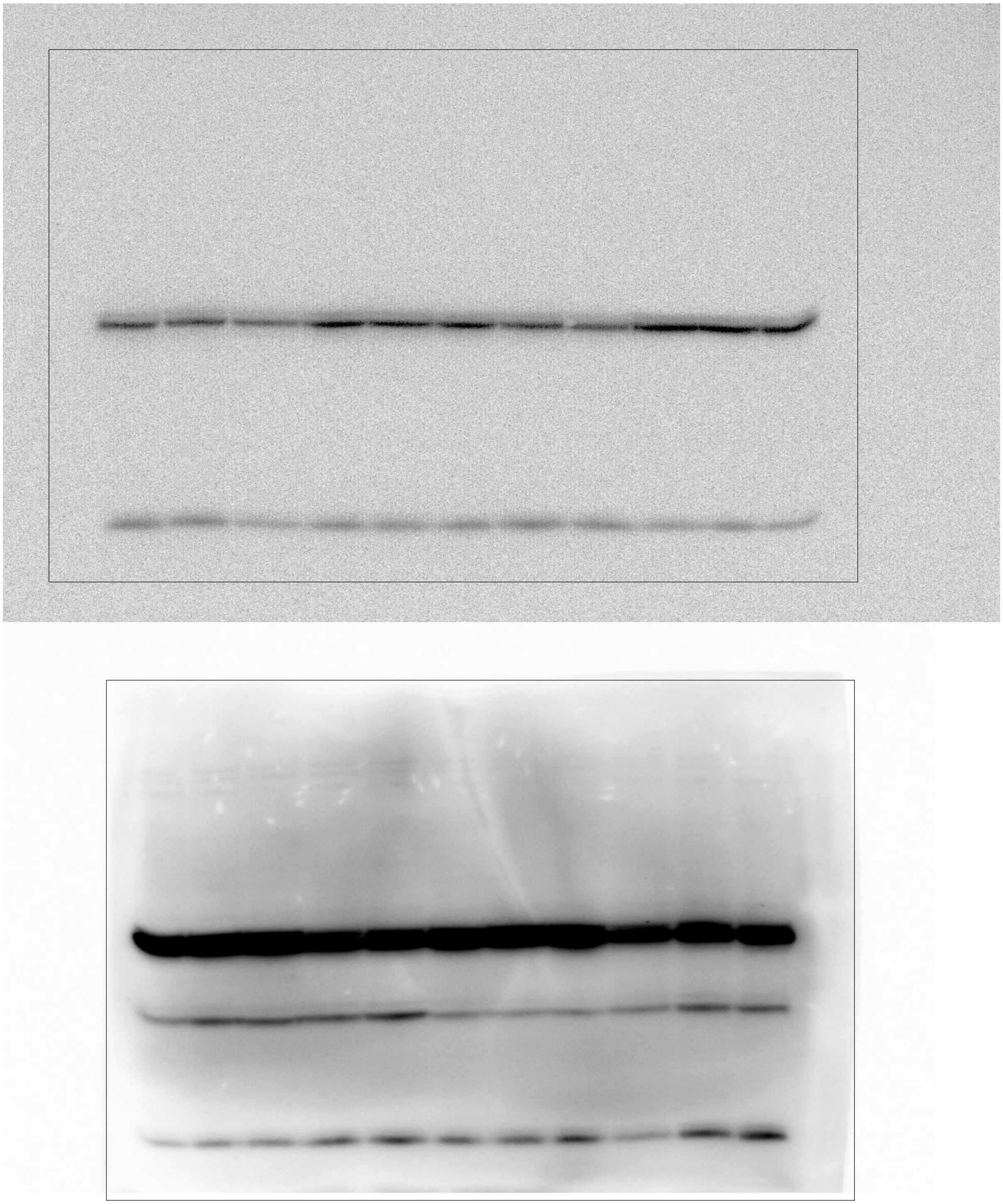

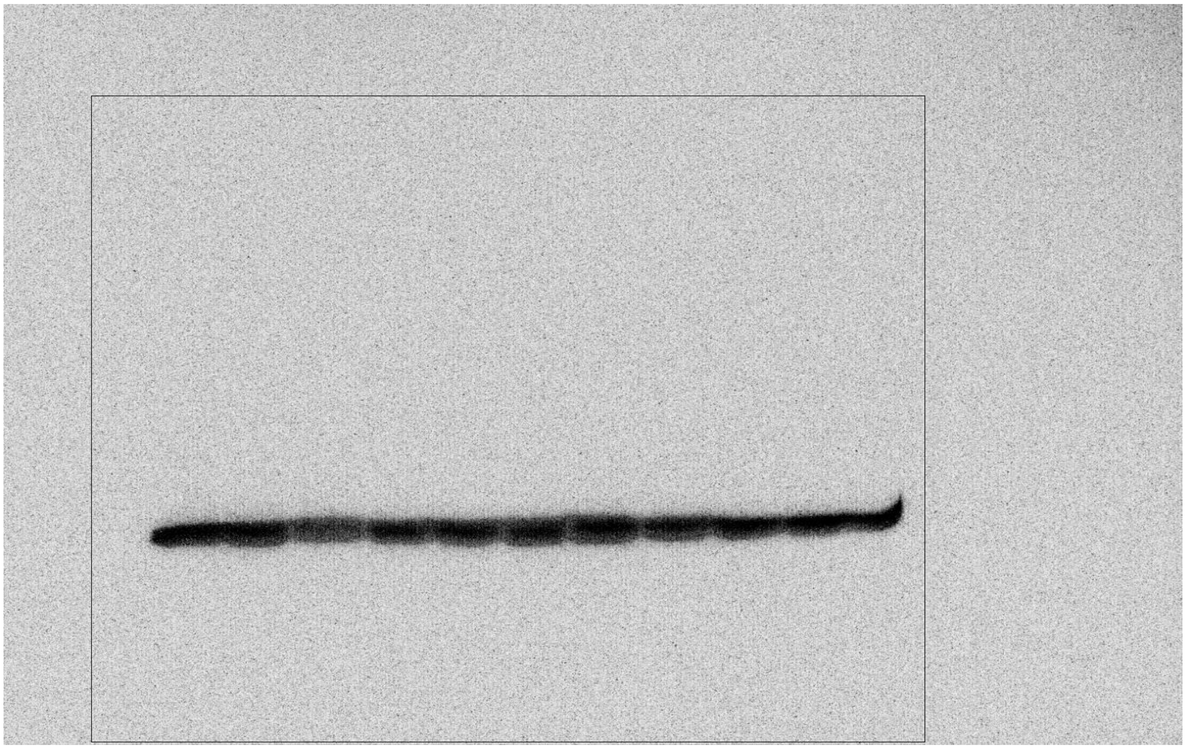

Figure 4-D

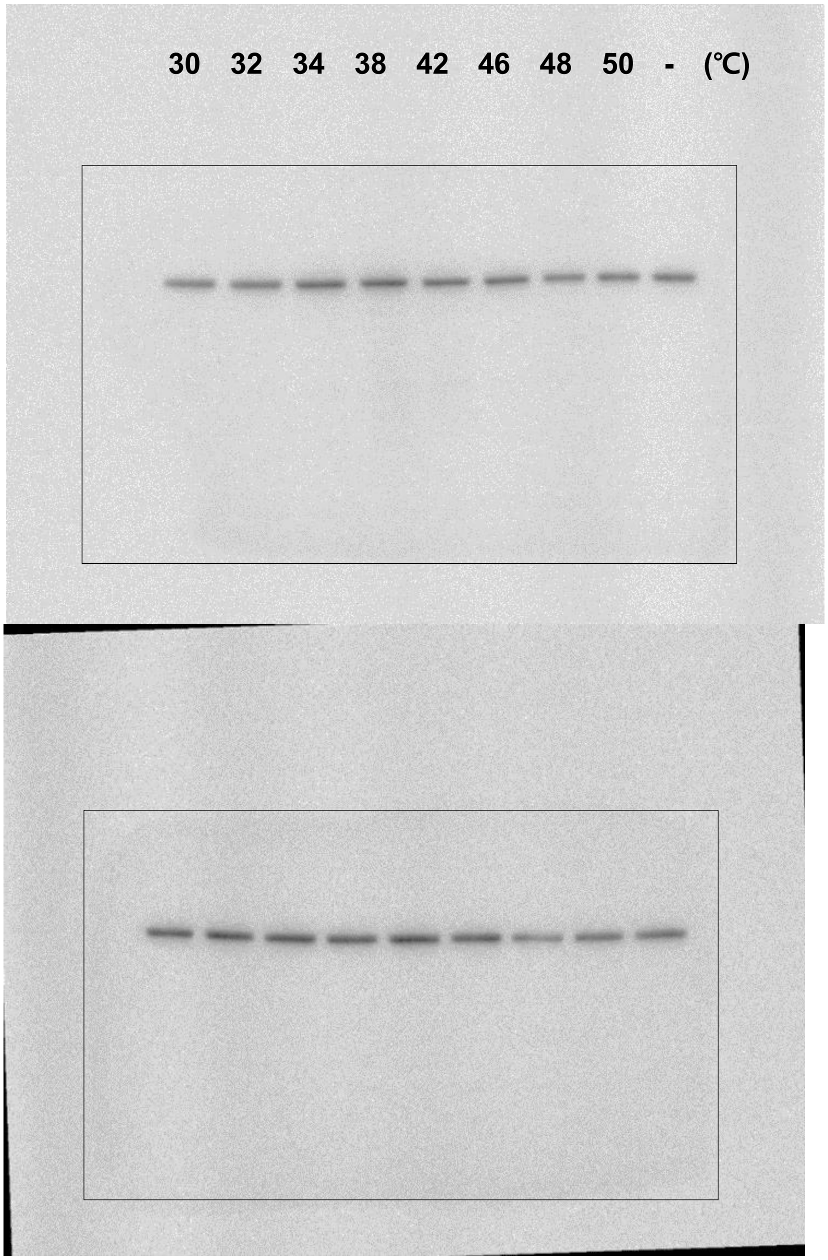

Figure 4-D

The upper membrane was cut after hybridization with CaN antibodies.

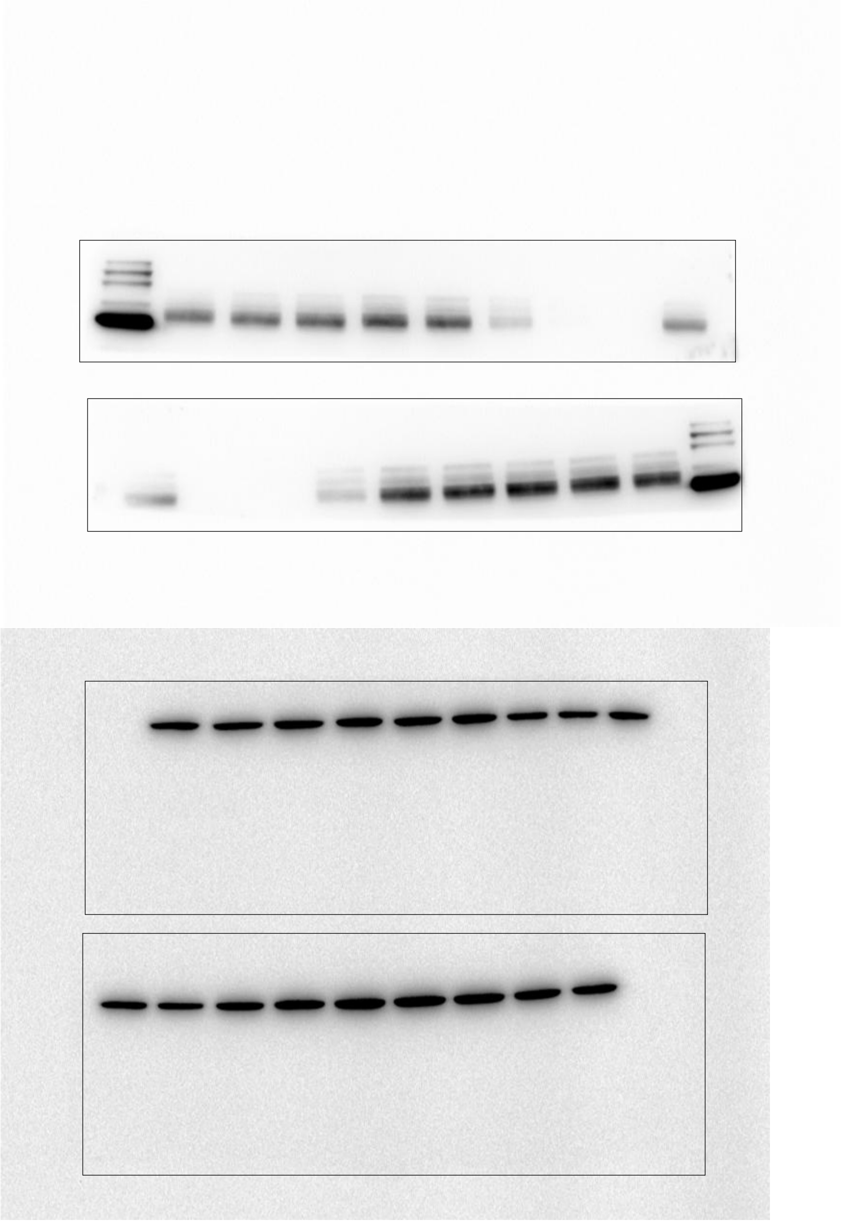

Supplementary figure

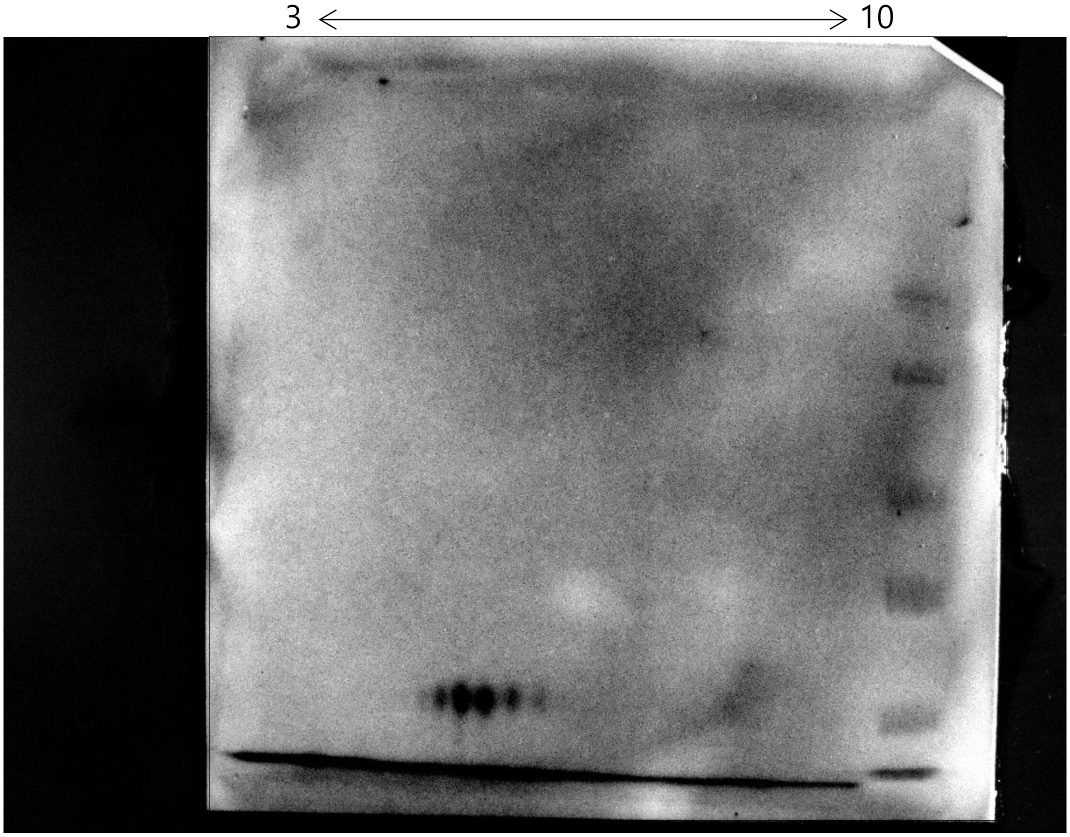

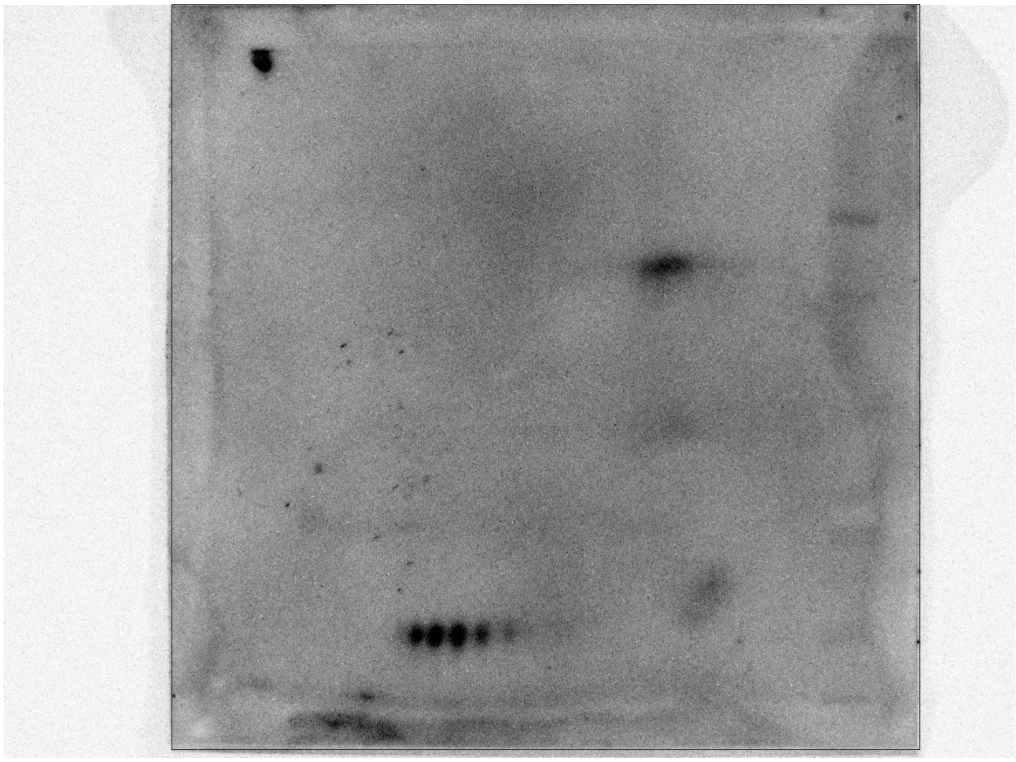

Supplement: Supplementary file 2 — Supplementary Material 2 [file 41598_2026_48882_MOESM2_ESM.pdf]
